# Assessment of groundwater quality and evaluation of scaling and corrosiveness potential of drinking water samples in villages of Chabahr city, Sistan and Baluchistan province in Iran

**DOI:** 10.1016/j.dib.2017.11.003

**Published:** 2017-11-08

**Authors:** Abbas Abbasnia, Mahmood Alimohammadi, Amir Hossein Mahvi, Ramin Nabizadeh, Mahmood Yousefi, Ali Akbar Mohammadi, Hassan Pasalari, Majid Mirzabeigi

**Affiliations:** a Department of Environmental Health Engineering, School of Public Health, Tehran University of Medical Science, Tehran, Iran; b Department of Environmental Health Engineering, Neyshabur University of Medical Sciences, Neyshabur, Iran; c Department of Environmental Health Engineering, School of Public Health, Iran University of Medical Science, Tehran, Iran

**Keywords:** Groundwater quality, WQI, Scaling and corrosiveness potential, GIS, Chabahr

## Abstract

The aims of this study were to assess and analysis of drinking water quality of Chabahar villages in Sistan and Baluchistan province by water quality index (WQI) and to investigate the water stability in subjected area. The results illustrated that the average values of LSI, RSI, PSI, LS, and AI was 0.5 (±0.34), 6.76 (±0.6), 6.50 (±0.99), 2.71 (±1.59), and 12.63 (±0.34), respectively. The calculation of WQI for groundwater samples indicated that 25% of the samples could be considered as excellent water, 50% of the samples were classified as good water category and 25% of the samples showed poor water category.

**Specifications Table**

**Table:** 

Subject area	Chemistry
More specific subject area	Describe narrower subject area
Type of data	Table, graph, figure
How data was acquired	All water samples were analyzed according to the Standard Methods for Examination of Water and Wastewater Temporary and permanent hardness, magnesium, calcium, and chloride were measured by titration method. The hydrogen ion concentration (pH) and electrical conductivity and opacity were analyzed with pH meter (model wtw, Esimetrwb) and turbidity meter (model Hach 50161/co 150 model P2100Hach, USA), respectively. Also, fluoride, nitrate, and sulfate were determined with Hach DR5000 spectrophotometer and compared with internal standards.
Data format	Raw, analyzed
Experimental factors	The mentioned parameters above, in abstract section, were analyzed according to the standards for water and wastewater treatment handbook.
Experimental features	The levels of physical and chemical parameters were determined.
Data source location	Chabahar, Sistan and Baluchistan province, Iran
Data accessibility	Data are included in this article

**Value of the data**

1. These data could be helpful for many organizations, such as rural water and wastewater organizations, water treatment plants, water resources management, and the Ministry of Energy, which need these to make decisions and adopt guidelines for water quality management.
2. The zoning of the scaling and corrosion indices and water quality index (WQI) was done to provide a clear picture of the water quality in the water resources at the villages of Chabahar.
3. In dry and semi-arid climates such as Iran, groundwater is almost the main source of water supply, therefore, the continuous monitoring of the quality of these valuable resources is very necessary.

## 1 Data

**Fig. 1:**
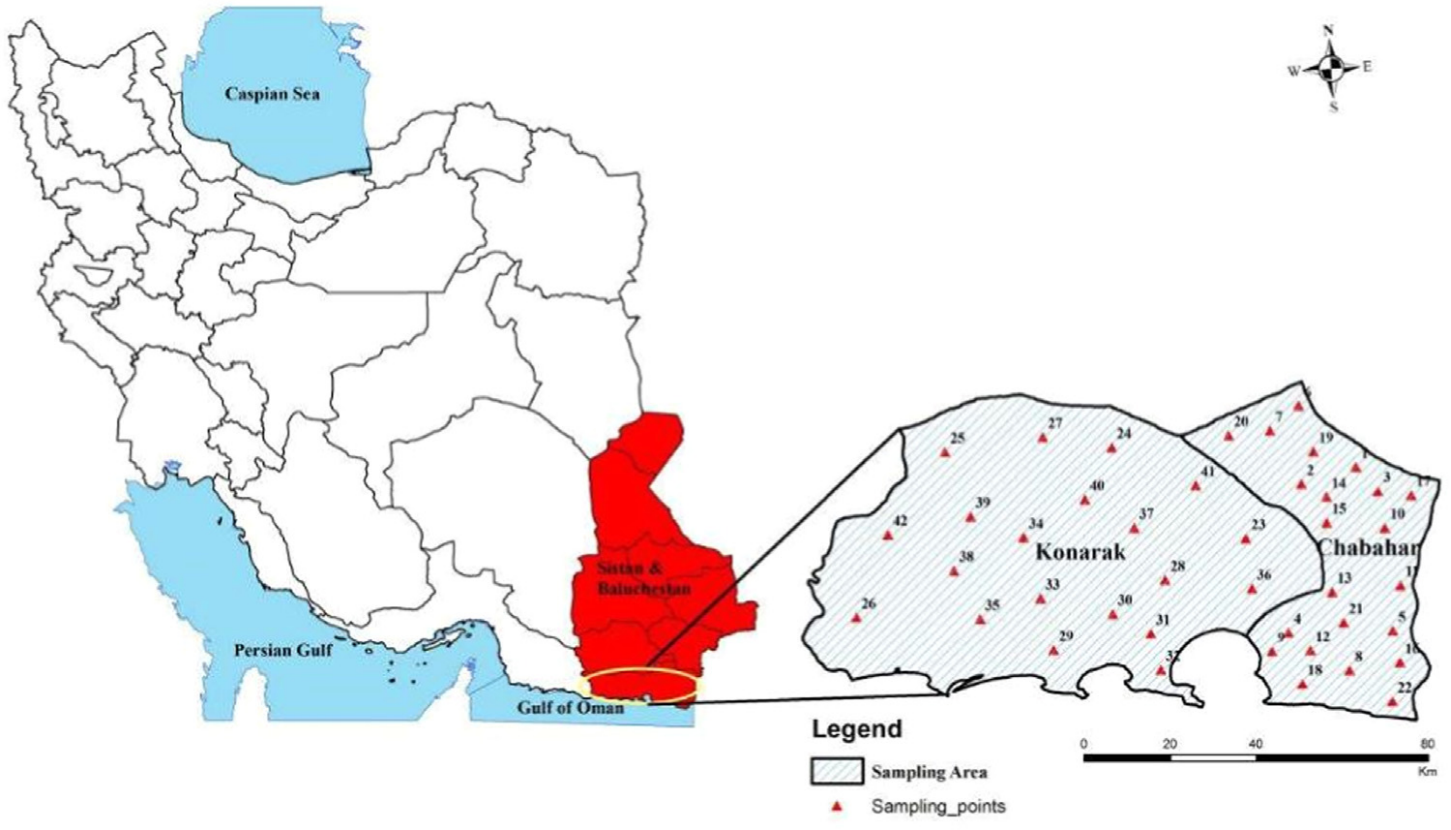
Location of water sampling sites in Chabahar city.

**Fig. 2:**
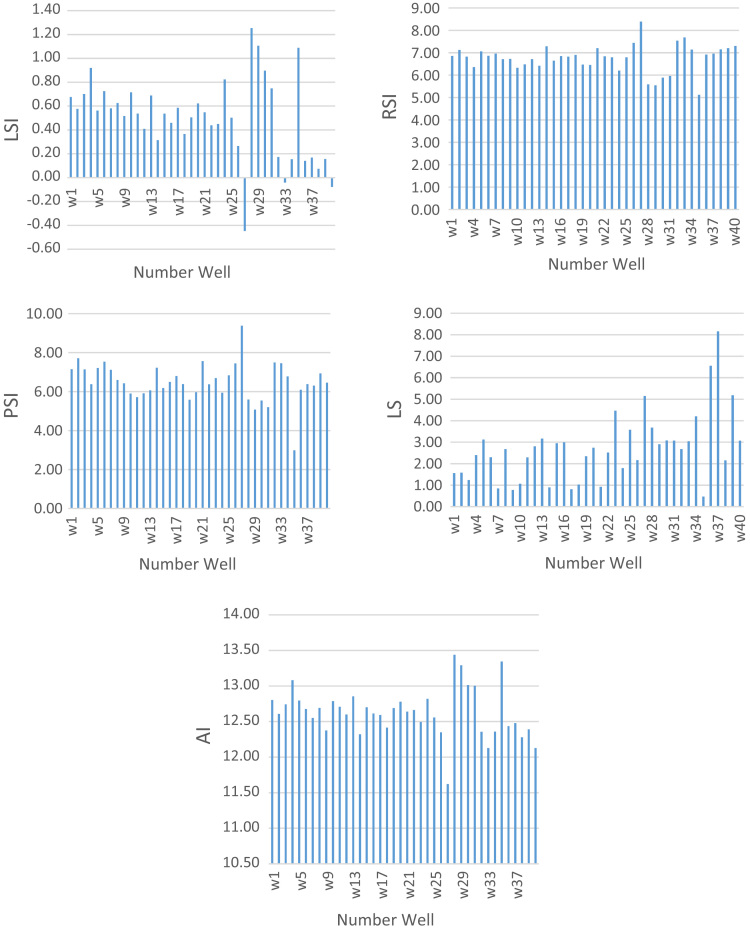
Spatial distribution of AI, LS, LSI, PSI, and RSI in region studied.

**Fig. 3:**
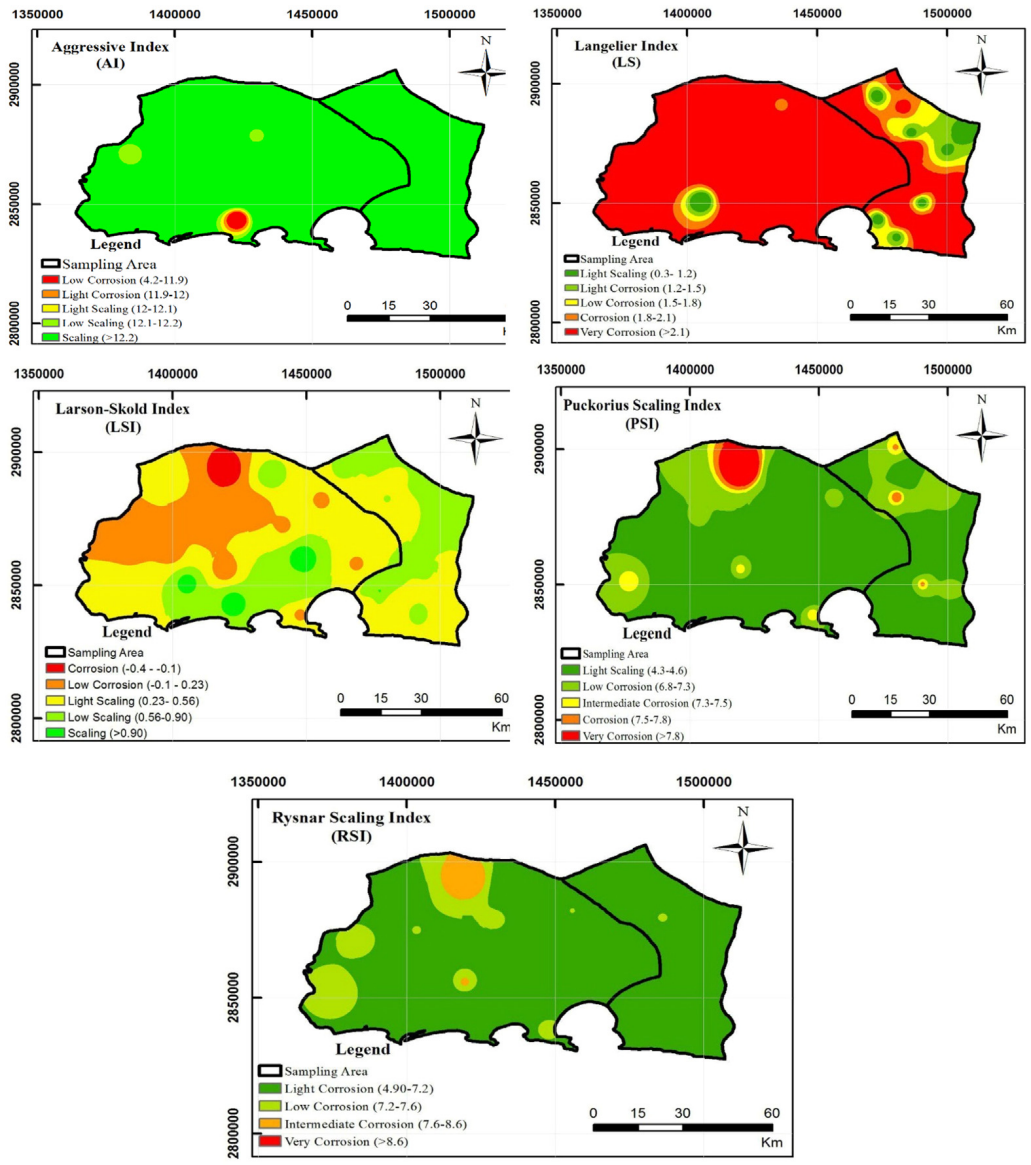
Spatial distribution of AI, LS, LSI, PSI, and RSI in region studied.

The parameters and indices were calculated in the experiments are including chloride ion, sulfate, temperature, Electrical Conductivity (EC), Total Dissolved Solids (TDS), pH, total alkalinity, bicarbonate ions, and calcium hardness according to standard methods for examination of water and wastewater [1]. Then LSI, RSI, PSI, LS, and AI were used to evaluate the water stability. Fig. 1 shows the sampling locations and Table 1 presents the indexes, equation, and some definition and criteria for categorizing the stability of the water. The chemical and physical properties of drinking water are presented in Table 2, Table 3. Table 4 shows the water stability indices in different parts of the region studied. As seen in Table 4, 7.5, 30, 80.72.5, and 97.5% of water supplies of Chabahar were corrosive according to the obtained results from LSI, RSI, LS, PSI, and AI, respectively (Fig. 2). Estimated corrosion indexes with GIS software are shown in Fig. 3. In the following we calculated water quality index (WQI).

**Table 1:** Corrosion and saturation indices, equation and criteria for categorizing the stability of the water used in the study [3], [4].

	Equation	Index value	Water condition
Langelier saturation	LSI=pH−pHs	LSI>0	Super saturated, tend to precipitate CaCO3
index (LSI)	pHs=A+B−log (Ca2+)−log	LSI=0	Saturated, CaCO3 is in equilibrium
	(Alk) pH<=9.3		
	pHs=(9.3+A+B)−(C+D)	LSI<0	Under saturated, tend to dissolve solid CaCO3
	(3) pH>9.3		
Ryznar stability	RSI=2pHs−pH	RSI<6	Super saturated, tend to precipitate CaCO3
index (RSI)		6<RSI<7	Saturated, CaCO3 is in equilibrium
		RSI>7	Under saturated, tend to dissolve solidCaCO3
Puckorius scaling	PSI=2 (pHeq)−pHs	PSI<6	Scaling is unlikely to occur
index (PSI)	pH=1.465+log	PSI>7	Likely to dissolve scale
	(T.ALK)+4.54		
	pHeq=1.465×log(T.ALK)+4.54		
Larson-skold index	Ls=(Cl–+SO42)/(HCO3+CO_3_^2^)	LS<0.8	Chloride and sulfate are unlikely to interfere with the
(LS)			formation of protecting film
		0.8<LS<1.2	Corrosion rates may be higher than expected
		LS>1.2	High rates of localized corrosion may be expected
Aggressive index	AI=pH+log[(Alk)(H)]	AI>12	Non aggressive
(AI)		10<AI<12	Moderately aggressive
		AI<10	Very aggressive

**Table 2:** Water quality characteristics associated with corrosion and scaling tendency.

**Number**	**ALK**	**CL**^**−**^	**SO**_**4**_^**2−**^	**Temp**	**EC**	**TDS**		**HCO**_**3**_^**−**^	**CaH**
**Well**	**mg/L CaCO3**	**(mg/L)**	**(mg/L)**	**°C**	**(μmhos/cm)**	**(mg/L)**	**pH**	**(mg/L)**	**mg/L CaCO3**
w1	200.08	222	90	19	1063	680.32	8.21	200.08	196
w2	141.52	174	50	22	845	540.8	8.28	141.52	150
w3	202.52	171	80	22	920	588.8	8.23	202.52	160
w4	305	363	370	22	2310	1478.4	8.2	305.00	250
w5	248.88	377	400	19	2320	1484.8	8.19	248.88	162
w6	129.32	197	100	26	927	593.28	8.31	129.32	180
w7	217.16	114	70	25	886	567.04	8.12	217.16	124
w8	263.52	286	420	26	2230	1427.2	7.97	263.52	200
w9	253.76	78	120	30	853	545.92	7.76	253.76	162
w10	312.32	184	150	23	1434	917.76	7.76	312.32	340
w11	390.4	505	390	23	2850	1824	7.56	390.4	360
w12	385.52	391	690	23	3250	2080	7.53	385.52	304
w13	295.24	375	560	23	2770	1772.8	7.8	295.24	384
w14	224.48	112	90	23	831	531.84	7.92	224.48	112
w15	307.44	398	510	23	2770	1772.8	7.72	307.44	312
w16	278.16	363	470	23	2540	1625.6	7.77	278.16	250
w17	241.56	86	110	23	835	534.4	8	241.56	162
w18	295.24	153	150	23	1197	766.08	7.64	295.24	202
w19	409.92	483	480	23	3140	2009.6	7.48	409.92	396
w20	312.32	347	510	23	2620	1676.8	7.7	312.32	384
w21	209.84	94	100	19	785	502.4	8.3	209.84	104
w22	307.44	264	510	19	2150	1376	7.72	307.44	284
w23	170.8	663	100	27	2240	1433.6	7.7	170.80	364
w24	273.28	242	250	27	1570	1004.8	7.85	273.28	340
w25	158.6	177	390	24	1495	956.8	7.8	158.60	360
w26	217.16	390	80	23	1556	995.84	7.97	217.16	110
w27	22.04	475	660	27	2660	1702.4	7.5	220.04	600
w28	270.84	197	800	22	2710	1734.4	8.1	270.84	806
w29	331.84	203	760	22	2690	1721.6	7.76	331.84	1024
w30	239.12	277	460	23	1973	1262.72	7.68	239.12	899.95
w31	326.96	206	800	19	2670	1708.8	7.46	326.96	1070
w32	209.84	362	200	19	1656	1059.84	7.89	209.84	140
w33	175.68	234	300	19	1481	947.84	7.6	175.68	192
w34	170.8	238	480	19	1896	1213.44	7.45	170.8	474
w35	2207.4	432	600	19	2680	1715.2	7.3	2207.4	502
w36	236.68	751	800	19	3450	2208	7.2	236.68	728
w37	190.32	727	825	19	3750	2400	7.3	190.32	790
w38	287.92	251	370	19	1915	1225.6	7.3	287.92	332
w39	165.92	360	500	19	2330	1491.2	7.52	165.92	448
w40	231.8	262	450	19	1920	1228.8	7.15	231.8	410
Mean	295.47	304.6	381.13	22.18	2004.2	1282.69	7.77	300.42	369.20
Min	22.04	78	50	19	785	502.4	7.15	129.32	104
Max	2207.4	751	825	30	3750	2400	8.31	2207.4	1070
St.dev.	319.32	163.96	244.12	2.87	827.10	529.34	0.32	316.50	254.51

**Table 3:** Statistics of groundwater parameters.

**Number**	**NO3**	**NO2**	**F**	**PO4**	**K**	**Na**	**Mg**	**Ca**	**TH**
**Well**	**(mg/L)**	**(mg/L)**	**(mg/L)**	**(mg/L)**	**(mg/L)**	**(mg/L)**	**(mg/L)**	**(mg/L)**	**(mg/L CaCO3)**
w1	2	0	0.2	0.17	6	117	15.84	78.4	260.99
w2	6.2	0	0.27	0.19	4	80	24	60	248.65
w3	3.5	0	0.47	0.1	6	90	34.08	64	300.15
w4	11.88	0.02	0.54	0.12	8	380	36	100	397.95
w5	10.6	0.05	0.8	0.16	8	390	29.28	64.8	282.38
w6	8.5	0	0.12	0.14	4	100	24.96	72	282.57
w7	8.5	0	0.28	0.06	4	105	20.64	49.6	208.85
w8	11	0	0.68	0.29	7	370	34.08	80	340.10
w9	9.3	0	0.33	0.15	4	90	24.96	64.8	264.59
w10	87.5	0.42	1.53	0.22	7	115	28.8	136	458.19
w11	11.5	0.02	0.83	0.06	7	390	48	144	557.23
w12	5.7	0.01	1.11	0.04	7	537	30.72	121.6	430.14
w13	11.5	0.02	0.63	0.05	7	426	37.44	153.6	537.72
w14	11	0.01	0.39	0.04	5	125	11.52	44.8	159.30
w15	9.3	0.02	0.87	0.05	6	435	37.92	124.8	467.78
w16	10	0.05	0.19	0.06	8	380	42.72	100	425.62
w17	10.5	0.02	0.25	0.04	4	85	21.12	64.8	248.78
w18	6.5	0.02	0.46	0.06	5	120	23.52	80.8	298.61
w19	11.5	0.02	0.33	0.03	7	440	74.88	158.4	703.88
w20	10.5	0.01	0.64	0.05	8	320	42.72	153.6	559.46
w21	4	0.01	1	0.1	6	116	12.96	41.6	157.24
w22	4.84	0	0.81	0.08	10	325	36.96	113.6	435.86
w23	19.4	0	0.5	0.22	7	260	52.32	145.6	579.02
w24	16.72	0.02	0.39	0.19	7	97	71.04	136	632.13
w25	17.2	0	0.93	0.25	6	130	28.8	144	478.17
w26	10	0.02	0.35	0.2	5	240	38.8	44	269.65
w27	14	0	0.63	0.16	6	260	52.8	240	816.71
w28	8	0.01	0.65	0.12	5	94	92.16	322.4	1184.55
w29	10.56	0.01	0.84	0.1	5	120	44.16	409.6	1204.62
w30	8.8	0	0.31	0.04	4	234	29.76	359.98	1021.42
w31	10.12	0.01	0.71	0.03	10	101	39.36	428	1230.80
w32	22.5	0.03	0.32	0.25	5	307	16.8	56	209.01
w33	10.5	0	0.49	0.26	6	225	22.08	76.8	282.70
w34	14	0	0.9	0.15	5	190	25.92	189.6	580.17
w35	13	0.01	0.51	0.06	8	330	48.48	200.8	701.04
w36	18	0.01	0.56	0.06	10	380	61.92	291.2	982.11
w37	8.5	0	0.5	0.01	11	440	67.2	316	1065.78
w38	11.44	0	0.42	0.02	7	248	34.08	132.8	471.94
w39	9.5	0	0.22	0.05	6	273	16.32	179.2	514.67
w40	7.5	0	0.32	0.36	8	210	26.4	164	518.22
Mean	12.39	0.02	0.56	0.12	6.48	241.88	36.54	147.68	519.22
Min	2	0	0.12	0.01	4	80	11.52	41.6	157.24
Max	87.5	0.42	1.53	0.36	11	537	92.16	428	1230.80
St.dev.	12.88	0.07	0.29	0.09	1.81	132.72	17.88	101.81	299.65

**Table 4:** Drinking water stability of Chabahar water distribution networks.

**Index**						**Water stability**				
**Number Well**	**LSI**	**RSI**	**LS**	**PSI**	**AI**	**AI**	**PSI**	**LS**	**RSI**	**LSI**
w1	0.68	6.86	1.56	7.16	12.80	NA	Ct	Ct	S	St
w2	0.58	7.13	1.58	7.72	12.61	NA	Ct	Ct	Ct	St
w3	0.70	6.83	1.24	7.14	12.74	NA	Ct	Ct	S	St
w4	0.92	6.36	2.40	6.38	13.08	NA	Ct	Ct	S	St
w5	0.56	7.07	3.12	7.21	12.80	NA	Ct	Ct	Ct	St
w6	0.73	6.86	2.30	7.54	12.68	NA	Ct	Ct	S	St
w7	0.58	6.96	0.85	7.11	12.55	NA	Ct	S	S	St
w8	0.63	6.72	2.68	6.60	12.69	NA	Ct	Ct	S	St
w9	0.52	6.73	0.78	6.42	12.37	NA	Ct	St	S	St
w10	0.71	6.33	1.07	5.90	12.79	NA	St	S	S	St
w11	0.54	6.48	2.29	5.71	12.71	NA	St	Ct	S	St
w12	0.41	6.71	2.80	5.91	12.60	NA	St	Ct	S	St
w13	0.69	6.42	3.17	6.06	12.85	NA	Ct	Ct	S	St
w14	0.31	7.29	0.90	7.23	12.32	NA	Ct	S	Ct	St
w15	0.54	6.65	2.95	6.18	12.70	NA	Ct	Ct	S	St
w16	0.46	6.85	2.99	6.50	12.61	NA	Ct	Ct	S	St
w17	0.59	6.83	0.81	6.80	12.59	NA	Ct	S	S	St
w18	0.37	6.91	1.03	6.39	12.42	NA	Ct	S	S	St
w19	0.51	6.47	2.35	5.58	12.69	NA	St	Ct	S	St
w20	0.62	6.46	2.74	5.96	12.78	NA	St	Ct	S	St
w21	0.55	7.20	0.92	7.56	12.64	NA	Ct	S	Ct	St
w22	0.44	6.84	2.52	6.38	12.66	NA	Ct	Ct	S	St
w23	0.45	6.80	4.47	6.69	12.49	NA	Ct	Ct	S	St
w24	0.82	6.20	1.80	5.94	12.82	NA	St	Ct	S	St
w25	0.50	6.80	3.58	6.83	12.56	NA	Ct	Ct	S	St
w26	0.27	7.44	2.16	7.45	12.35	NA	Ct	Ct	Ct	St
w27	−0.45	8.39	5.92	9.39	11.62	MA	Ct	Ct	Ct	Ct
w28	1.25	5.59	3.68	5.59	13.44	NA	St	Ct	St	St
w29	1.11	5.55	2.90	5.07	13.29	NA	St	Ct	St	St
w30	0.90	5.89	3.08	5.54	13.01	NA	St	Ct	St	St
w31	0.75	5.96	3.08	5.20	13.00	NA	St	Ct	St	St
w32	0.17	7.54	2.68	7.49	12.36	NA	Ct	Ct	Ct	St
w33	−0.04	7.68	3.04	7.45	12.13	NA	Ct	Ct	Ct	Ct
w34	0.15	7.14	4.20	6.78	12.36	NA	Ct	Ct	Ct	St
w35	1.09	5.12	0.47	2.98	13.34	NA	St	St	St	St
w36	0.14	6.92	6.55	6.10	12.44	NA	Ct	Ct	S	St
w37	0.17	6.96	8.15	6.38	12.48	NA	Ct	Ct	S	St
w38	0.08	7.15	2.16	6.31	12.28	NA	Ct	Ct	Ct	St
w39	0.16	7.21	5.18	6.93	12.39	NA	Ct	Ct	Ct	St
w40	−0.08	7.31	3.07	6.45	12.13	NA	Ct	Ct	Ct	Ct
**Ct**	7.5	30	80	72.5	97.5					
**Stable**	0	57.5	15	0	0					
**St**	92.5	12.5	5	27.5	2.5					
**Mean**	0.5	6.76	6.50	2.71	12.63					
**Max**	1.25	8.39	9.39	8.15	13.44					
**Min**	−0.45	5.12	2.98	0.47	11.62					
**St.dev**	0.34	0.60	0.99	1.59	0.34					

**Fig. 4:**
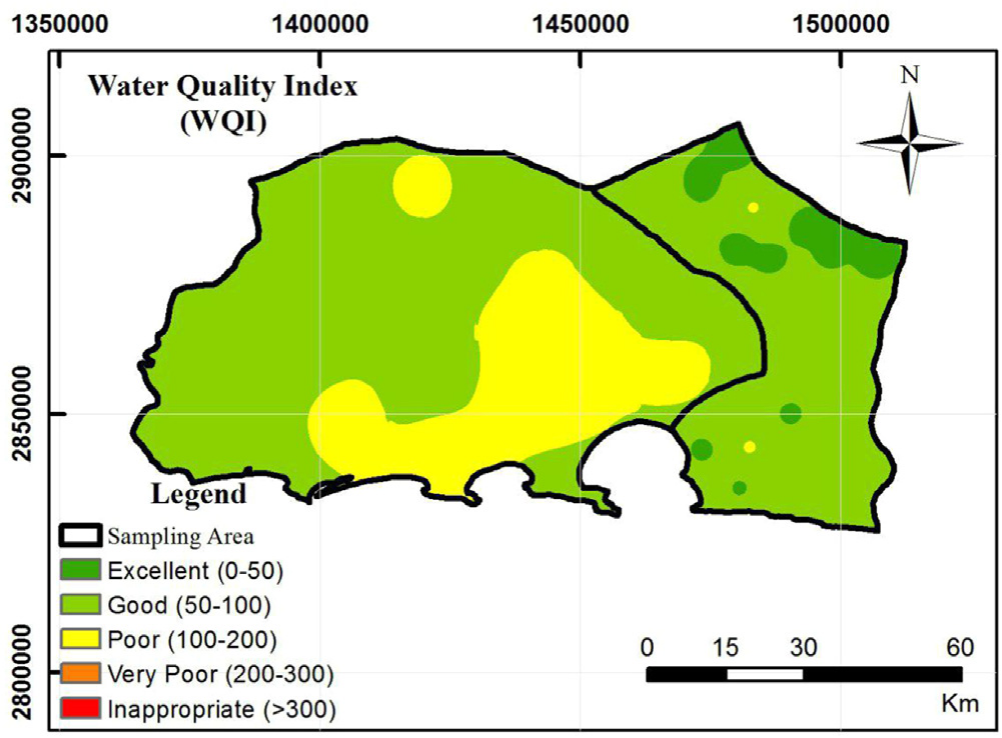
Spatial distribution map of water quality index.

An important parameter for determining the water quality and its sustainability for drinking purposes is water quality index (WQI). In order to provide the composite influence of individual water quality parameters on the overall water quality WQI could be useful [2]. Also according to World Health Organization(WHO) 2011 standards calculating the WQI has been considered for drinking water quality assessment. The relative weight (Wi) was assigned for water quality parameters based on their relative importance on water quality for drinking purposes (Table 5). The water quality classification based on WQI values is shown in Table 6. The calculation of WQI for groundwater samples is shown in Table 7. A total of 40 samples were analyzed for WQI. Among these, 25% of the samples showed excellent water, 50% of the samples fell under good water category and 25% of the samples showed poor water category respectively (Fig. 4).

**Table 5:** Relative weight of chemical of physico-chemical parameters [5].

**Number**	**Factor**	**Factor Weight**	**WHO Standard**
1	K	2	12
2	Na	3	200
3	Mg	2	50
4	Ca	3	75
5	PO_4_	1	0.5
6	HCO_3_	2	500
7	NO_3_	5	45
8	NO_2_	5	3
9	SO_4_	4	250
10	CL	3	250
11	F	4	1.5
12	TH	3	6.5–8.2
13	EC	3	6.5–8.3
14	TDS	5	6.5–8.4
15	pH	3	6.5–8.5

**Table 6:** Water quality classification ranges and types of water based on WQI values [6].

**WQI value**	**Class**	**Explanation**
<50	Excellent	Good for human health
50–100	Good	Fit for human consumption
100–200	Poor	Water not in good condition
200–300	Very poor	Need attention before use
>300	Inappropriate	Need too much attention

**Table 7:** Water quality index (WQI) classification for individual samples.

**Number Well**	**WQI**	**Water quality rating**
W1	42.49	Excellent
W2	37.23	Excellent
W3	42.44	Excellent
W4	80.74	Good
W5	77.94	Good
W6	41.77	Excellent
W7	35.86	Excellent
W8	77.42	Good
W9	40.07	Excellent
W10	89.45	Good
W11	95.58	Good
W12	103.82	Poor
W13	97.31	Good
W14	36.89	Excellent
W15	93.08	Good
W16	82.16	Good
W17	38.52	Excellent
W18	47.05	Excellent
W19	103.38	Poor
W20	91.60	Good
W21	39.24	Excellent
W22	83.33	Good
W23	83.40	Good
W24	72.00	Good
W25	73.46	Good
W26	53.74	Good
W27	110.21	Poor
W28	119.88	Poor
W29	125.81	Poor
W30	105.36	Poor
W31	128.97	Poor
W32	61.70	Good
W33	60.05	Good
W34	84.75	Good
W35	124.02	Poor
W36	140.84	Poor
W37	144.35	Poor
W38	73.74	Good
W39	83.48	Good
W40	78.55	Good

## 2 Experimental design, materials and methods

### 2.1 Study area description

Chabahar city in Sistan and Baluchistan province and its aquifers are located in South-East Iran between the latitudes 25°17′ N and longitudes 60°37′ E, encompassing an area of about 9739 km^2^ (Fig. 1). The study area is a semi-flat plain region with a gentle slope toward the south has a warm, temperate climate. Also the air's highest and lowest temperatures are 50 °C and −7 °C, respectively, with an annual average of 25 °C. The subjected area was classified as a semiarid, and the precipitation range is 70–130 mm per year with the evaporation rate of 4000 mm per year (four times as high as Iran's average) [7].

### 2.2 Sample collection and analytical procedures

In this cross-sectional study, 40 rural drinking water sources in Chabahar villages in Sistan and Baluchistan province were analyzed during 12 months (2010–2011) according to physical and chemical parameters. Fig. 1 shows the study area and sampling locations in this study. Samples were collected in polythene bottles (1L) and then immediately transported at 4°C to the central laboratory of the water and wastewater company. All water samples were analyzed according to the Standard Methods for Examination of Water and Wastewater and using titration method permanent hardness, magnesium, calcium, and chloride were measured [1]. The concentration of hydrogen ion (pH) and electrical conductivity and opacity were also analyzed with pH meter (model wtw, Esimetrwb) and turbidity meter (model Hach 50161/co 150 model P2100Hach, USA), respectively. On the other hand, using Hach DR5000 spectrophotometer fluoride, nitrate, and sulfate were determined compared with internal standards [1], [8], [9], [10], [11]. Then, to calculate WQI, the weight for physical and chemical parameters were determined with respect to the relative importance of the overall water quality for drinking water purposes, as shown in Table 6, Table 7 and the Langelier saturation index, Ryznar saturation index, Aggressiveness index, Larson–Skold index, and Puckorius scaling index were calculated and the results were classified in three categories: scaling, stabilized, and corrosive. Table 1 presents the indices, equations and some definitions and criteria for categorizing the stability of the water. Finally, the severity of corrosion in different water supply systems of Chabahar villages in Sistan and Baluchistan province was displayed using a geographic information system (GIS). All analyses were done using Excel 2010 and Arc GIS 10.3 software.

#### 2.2.1 Water quality index calculation

To calculate the WQI, the weight for the physico-chemical parameters were assigned according to the relative importance of parameters in the overall quality of water for drinking purposes.

Using the following equation, the relative weight was computed:

$$\mathrm{Wi}=\sum \frac{\mathrm{Wi}}{\sum_{i=1}^{n}\mathrm{Wi}}$$

where

*Wi* is the relative weight
*Wi* is the weight of each parameter
*n* is the number of parameters.

The quality rating scale for each parameter is calculated by dividing its concentration in each water sample by its respective standards (World Health Organization 2011 [5]) and multiplied the results by 100.

$$\mathrm{qi}=(\frac{\mathrm{Ci}}{\mathrm{Si}})\times 100$$

where

*qi* is the quality rating
*Ci* is the concentration of each chemical parameter in each sample in milligrams per liter
*Si* is the World Health Organization standard for each
Chemical parameter in milligrams per liter according to the guidelines of the (WHO 2011 [5])

For computing the final stage of WQI, the SI is first determined for each parameter. The sum of SI values gives the water quality index for each sample.

Si=Wi×qi

WQI=∑SIi

where

*SIi* is the sub-index of it parameter
*qi* is the rating based on concentration of it parameter
*n* is the number of parameters [2]
