# Supplementary material for: Assessment of groundwater quality and evaluation of scaling and corrosiveness potential of drinking water samples in villages of Chabahr city, Sistan and Baluchistan province in Iran
Source: Data Brief. 2017 Nov 8;16:182–92. doi: 10.1016/j.dib.2017.11.003 (PMC5702867; doi:10.1016/j.dib.2017.11.003)
Supplement: Supplementary file 1 — Transparency document [file mmc1.docx]

Conflict of Interest

The authors of this article declare that they have no conflict of interests.
